# The Bright and Enlightening Science of Quantum Dots

**DOI:** 10.1021/acs.nanolett.3c03904

**Published:** 2023-10-23

**Authors:** Liberato Manna

**Affiliations:** Nanochemistry Department, Istituto Italiano di Tecnologia, Via Morego 30, 16163 Genova, Italy

**Keywords:** quantum dots, nanocrystals, colloidal chemistry, synthesis, quantum confinement

## Abstract

The 2023 Nobel Prize in Chemistry was awarded to Alexei Ekimov, Louis Brus, and Moungi Bawendi for the discovery and development of quantum dots, an area of research ripe with exciting results in terms of both fundamental science and present and forthcoming applications. Quantum dots, with their colors and their intriguing properties, have fascinated and engaged generations of scientists over the last 40 years, including myself. I present here a brief historical perspective of the field, from my personal standpoint and with insights from my own career, along with an outlook on what I believe will be the most interesting future developments in the field.

The news of the 2023 Nobel Prize in Chemistry for quantum dots has galvanized the colloidal chemistry community worldwide. These recognitions were long awaited: quantum dots are perhaps some of the most vivid and spectacular examples of how matter is governed by the laws of quantum mechanics and represent the simplest and most practical demonstration of the particle in a box problem that science students are familiar with. They are arguably the most compelling example of a functional nanotechnology product entirely delivered by chemical synthesis that has found its way into applications. How has all of this started?

Back in the late 1970s to early 1980s, Alexei Ekimov, then working in the former Soviet Union, was studying copper chloride nanocrystals which he grew in glassy matrixes and found that the optical absorption properties of these nanocrystals were dependent on their size.^1^ Deducing such a relationship was not so straightforward with the limited tools of the epoch. Stimulated by Ekimov’s discovery, Alexander Efros, also from the Soviet Union, developed the general theory describing the influence of size quantization on the optical absorption for a broad class of semiconductors.^2^ Independently, Louis Brus, back then at Bell Laboratories in the United States, also noticed quantization effects in light absorption but in cadmium sulfide nanocrystals grown as colloidal suspensions^3^ and developed also basic theoretical models to describe them.^4,5^

These developments by such groups and by others (for example, by Henglein^6^ and co-workers) were great and exciting news, and a robust theory continued to be developed in parallel to both corroborate and predict the physics emerging from confined systems. Studies on nanocrystals grown in glassy matrixes (the first observations of quantum confinement in what later became popular materials such as CdSe and PbSe were indeed made on these types of samples) and in colloidal suspensions continued to capture the attention of an increasing number of groups. Another promising direction was emerging at the time. This came from the ability to synthesize, in the liquid phase, large inorganic semiconductor clusters with a precise number of “core” atoms and surface ligand molecules, which could be characterized with a high level of detail because it was possible to crystallize them into crystals of high enough quality for their structure to be solved from X-ray diffraction data.^7,8^ These large clusters, being already some nanometers in size, bridged the gap between ultrasmall clusters of only a few atoms and the (not precisely defined) colloidal nanocrystals made by Brus, Henglein, and others. This area of research later developed into the interesting field of semiconductor “magic size” clusters, very active nowadays.^9^

An important milestone for the wet chemistry of quantum dots came in 1993 when Christopher Murray, David Norris and Moungi Bawendi, partly building on chemistry developed by Michael L. Steigerwald and others, published a seminal work^10^ in the *Journal of the American Chemical Society* demonstrating how colloidal nanocrystals of cadmium chalcogenides could be grown with an unprecedented level of control by a hot injection method, consisting of rapidly mixing chemical precursors in a heated mixture of surfactants. The high temperature ensured the decomposition of the precursors and their transformation into monomers, triggering the nucleation of the nanocrystals. Concurrently, the dynamic environment promoted continuous binding and unbinding of the surfactants from the nanocrystal surface, enabling the sequential addition of monomeric species and leading to steady accretion of the nanocrystals, layer by layer. The method worked under water-free conditions and, also thanks to the high synthesis temperature, delivered highly crystalline quantum dots with low defect densities, which translated into superior optical properties compared to previous approaches. Additionally, the ligand shell around the nanocrystals guaranteed optimal stability once the reaction was quenched by cooling to room temperature.

The disarming simplicity of the method, together with the narrow size distribution and the distinct, beautifully size-dependent optical properties of the resulting colloidal dots, would eventually redirect dozens of groups worldwide to this exciting research direction. With such unique samples, scientists could better address very fundamental questions, above all how physical properties and transformations in solids depend on size^11^ and how they differ from the bulk, an area where Paul Alivisatos’ group at UC Berkeley made important discoveries (for example, in solid–solid phase transitions).^12^ These latter developments truly sparked my interest in the field in the late 1990s, fresh from my undergraduate studies in physical chemistry and crystallography and at the beginning of my Ph.D., and encouraged me to contact Alivisatos (my personal hero and inspiring mentor), who, to my great joy, admitted me in his group as a visiting student.

Soon it became possible to grow one or more epitaxial “shells” of materials on a nanocrystal “core” to create heterostructured nanocrystals in which the confinement of carriers could be finely engineered, and the efficiency of the emitted light would be quickly pushed to basically near unity.^13−15^ The synthesis of core/shells had already been attempted in the 1980s, for example, using aqueous approaches,^16^ but this new chemistry was much more controllable. The nucleation and growth processes were better scrutinized, and it was more clearly understood which molecules were responsible for tuning the surface reactivity of the nanocrystals and how this depended on crystal structure and on surface details. Such endeavors in later years enabled shape control, resulting in nanocrystals with shapes of various forms like rods,^17^ platelets,^18^ branched structures, and several others. These distinct shapes endowed the nanocrystals with unique properties, such as polarized emission, reduced overlap between absorbing and emitting states, and narrow emission line widths. Additionally, it provided fresh insight into how many-particle states, such as excitons, biexcitons, trions, and others, behave in these materials. At the beginning of my career, I was very lucky to be part of this initial “fever”, which in the early years focused on CdSe as a flagship material. In 1999, under the guidance of Alivisatos, together with Xiaogang Peng we discovered that certain surfactants (alkyl phosphonic acids) could be used to control the shape of CdSe nanocrystals and generate what we then called “quantum rods”,^17^ after which we went on to develop tetrapod shapes.

This is not just blue-sky research but also has strong implications for applications. All of these shapes became essentially testing grounds for assessing more refined theories of quantum confinement and for applications. Quantum rods, for example, when carefully aligned over large areas, have been used to fabricate polarized displays. Understanding and tuning the reactivity of nanocrystals has become another prolific field of research, with groups being able to grow nanocrystals made of multiple sections of different materials and to control the flow of carriers in the different sections. Ion exchange reactions (essentially the same reactions at work in ion exchange resins for water purification and in the fossilization of biological tissues) were used to “transmute” nanocrystals of a given material into another one while preserving their size and shape.^19^

In parallel with the synthesis, the enormous potential of colloidal quantum dots was also recognized in fields such as bioimaging, labeling, and sensing,^20,21^ light emitting diodes,^22^ solar cells,^23,24^ lasers,^25,26^ detectors,^27^ and luminescent solar concentrators.^28^ The basic features of the colloidal synthesis approach of Bawendi’s group were quickly harnessed to grow colloidal nanocrystals of other materials, such as group IV, IV–VI, and III–V semiconductors, metal oxides, and more, greatly impacting also other fields of fundamental research and applications. InP based quantum dots synthesized by modification of this methodology, offering a system that is fully compatible with the regulations on hazardous materials for electronic devices (ROHS), were essential for the wide-scale implementation in displays.

The techniques developed and refined on these materials were then readily available and put to good use when metal halide perovskites emerged (still a very hot topic today). These were first recognized for their exceptional performance as solid-state materials in solar cells, delivering record efficiency values, but subsequent pioneering research^29,30^ revealed that colloidal nanocrystals of lead halide perovskites were remarkable light emitters and were less affected by crystal defects or surface imperfections compared to the more established quantum dots. One should mention that CsPbBr_3_ quantum dots, in the form of thermally nucleated inclusions in CsBr crystals, had been known since 2001.^31^ Although quantum confinement is less important for the optical properties of these materials, adjusting the ionic composition (especially the halide ions) offers an equally effective strategy for tuning the wavelengths of absorption and emission,^32,33^ thereby revealing novel pathways to modulate their optoelectronic properties.

My career as a scientist in this field has been influenced by many towering figures. As a student, I eagerly read works by Ekimov, Brus, Efros, Bawendi, Nozik, Murray, Norris, Weller, Woggon, Gaponenko, Guyot-Sionnest, and many others. As a postdoc and then as an independent researcher, I witnessed the field’s rapid expansion. With so many rising stars in addition to the more consolidated groups, astounding results were being delivered daily. Essentially, every morning brought exciting news that one had to review just to catch up.

Nowadays, the most remarkable and widespread applications of colloidal quantum dots are in light emitting diodes and displays (the QLED TV sets indeed use colloidal quantum dots) and biological labeling, but before long, other applications may find their way to the market. Quantum dots have been postulated to be part of next-generation lasers, and a real breakthrough came this year with the demonstration of an electrically pumped colloidal quantum dot laser by Klimov’s group.^34^ Finding ways to embed quantum dots in a matrix and solving all the issues of sample degradation, aggregation, and light losses or preservation of quantum confinement behavior while allowing charge carriers to enter/exit the nanocrystal by careful surface chemistry are all examples of efforts that may soon lead to commercial applications such as efficient detectors, luminescent solar concentrators, and scintillators, especially since, in the latter case, the matrix can be of a plastic material that is moldable into any arbitrary shape.

Biomedical applications may also progressively benefit from this field. Quantum dots, by virtue of their fluorescence and surface functionalization with specific markers, may help surgeons to distinguish healthy from cancerous tissues. Here we will get another boost when near-infrared (NIR) quantum dots with low toxicity will be further developed, with potential applications such as subcutaneous and deep tissue imaging and photothermal therapy. Other widespread NIR applications will be in solar cells, medical applications, night vision and imaging in the short-wave IR range, facial recognition, and telecommunications. Quantum dots can emit one single photon at a time,^35^ a feature that many groups have studied in detail and that might lead to interesting developments in quantum technologies. Bawendi’s group recently demonstrated that single photons emitted from CsPbBr_3_ nanocrystals at low temperature are indistinguishable^36^ (with strong implications for quantum computing, quantum teleportation, and quantum key distribution), and single photons emitted from InP quantum dots on the other hand have a relatively long coherence time^37^ (a prerequisite for quantum technologies). Quantum dots have also been named as “artificial atoms”, due to their distinct atomic-like electronic states.^38^ Understanding how these states can be prepared and manipulated will be the next challenge to master these upcoming technologies. Chemical synthesis methods that controllably lead to dimers or more complex groupings of quantum dots intimately glued to each other are already delivering the first examples of “nanocrystal molecules”, with proof of inter-nanocrystal coupling generating bonding and antibonding states, similarly to molecules made of atoms.^39^

The ability to prepare quantum dots with close to monodispersity in size has been quickly harnessed to prepare ordered assemblies of a very large number of dots,^40^ essentially the equivalent of crystals but with nanocrystals instead of atoms or molecules as the fundamental components, with beautiful examples of superstructures. Akin to regular crystals, such assemblies can display collective properties arising from coupling of the individual nanocrystals, such as band-like transport^41^ or superfluorescence.^42^ These features can then be used for advanced applications. For example, superfluorescence can be exploited, in principle, to create a natural random number generator, and assemblies can be put to use as physical “simulators” that can be probed with optical tools to test collective solid state physics phenomena. The real challenge here is to fabricate new types of artificial solids with properties that cannot be obtained with ordinary crystals made of atoms or molecules.

All of these fascinating developments showcase the excitation and dynamics driving the field of quantum dot research since its early days and well into the future. This is possible first and foremost because chemists can devise new ingenious methods to synthesize quantum dots and tune their surface properties and their assembly behavior, providing new building blocks on which more refined experiments can be made. Still, many challenges lie ahead. The nucleation and growth of colloidal nanocrystals, together with the many chemical reactions involved in these processes, for example, still need to be investigated in greater detail, with more refined models emerging only recently. The chemistry and physics of the nanocrystal’s surface, defects, and ligand binding are adequately known only for a handful of cases, and various classes of quantum dots still need satisfactory syntheses. Take NIR III–V materials, such as InAs or InSb. These are of immense value for applications in telecommunication, and the best quality quantum dots of these materials have been grown with expensive and difficult-to-upscale physical deposition methods such as molecular beam epitaxy or metal–organic chemical vapor deposition. Even though the importance and the need to grow colloidal nanocrystals out of these materials was clear from the start,^43^ the chemistry used in their colloidal synthesis has made little progress. One can identify two reasons for this. First, the chemistry is indeed difficult, and the molecules involved are either too reactive or not reactive enough (and often highly toxic). Second, probing samples in the NIR region requires more expensive optical tools that only a few groups can afford. In this area, we are in urgent need of new precursors, new ligands, new redox agents, and more affordable optical tools. Also, we must understand the growth processes in more detail. Furthermore, we need new creative synthesis approaches, such as the ones Talapin’s group is developing using molten salts environments.^44^ Quantum dots of metal halides, of which halide perovskites are a subclass, are another fast growing research direction. These materials are highly labile, and consistent methods must be developed to improve their stability and grow epitaxial interfaces on them to master the same level of wave function engineering that is possible in nanocrystals of the more established materials. Interesting (and revealing) enough, it was indeed on a metal halide (copper chloride) that Ekimov first proved size dependent optical properties. This, along with the inspiring early works of the solution-based quantum dot pioneers Brus and Bawendi, can be taken as a reminder for the younger generations that, often, good old articles age very well and can acquire a new taste, and so they should not be overlooked. Sometimes, they are even awarded a Nobel Prize!
